# mzPeak: Designing a Scalable, Interoperable, and Future-Ready Mass Spectrometry Data Format

**DOI:** 10.1021/acs.jproteome.5c00435

**Published:** 2025-10-02

**Authors:** Tim Van Den Bossche, Theodore Alexandrov, Aivett Bilbao, Wout Bittremieux, Federico Ivan Brigante, Matthew Chase Chambers, Joshua Charkow, Eric Deutsch, Andrew W. Dowsey, Yasin El Abiead, Ralf Gabriels, Helge Hecht, Steffen Heuckeroth, Joshua A. Klein, Michael Knierman, Lennart Martens, Robert L. Moritz, Laura-Isobel McCall, Steffen Neumann, Yasset Perez-Riverol, Hannes L. Röst, Elliott J. Price, Jim Shofstahl, David L. Tabb, Julian Uszkoreit, Juan Antonio Vizcaíno, Mingxun Wang, Sander Willems, Dirk Winkelhardt, Oliver Kohlbacher, Samuel P. Wein

**Affiliations:** † Department of Biomolecular Medicine, Faculty of Medicine and Health Sciences, 26656 Ghent University, 9052 Ghent, Belgium; ‡ CompOmics, VIB—UGent Center for Medical Biotechnology, 82219 VIB, 9052 Ghent, Belgium; § Department of Pharmacology, 8784 University of California San Diego, La Jolla, California 92093, United States; ∥ Department of Bioengineering, University of California San Diego, La Jolla, California 92093, United States; ⊥ Environmental Molecular Sciences Laboratory, 6865 Pacific Northwest National Laboratory, Richland, Washington 99352, United States; # US Department of Energy Agile BioFoundry, Emeryville, California 94608, United States; ∇ University of Antwerp, 2020 Antwerpen, Belgium; ○ Institute of Organic Chemistry and Biochemistry of the Czech Academy of Sciences, 160 00 Prague, Czech Republic; ◆ University of Washington, Seattle, Washington 98195, United States; ¶ Donnelly Centre for Cellular and Biomolecular Research, 7938 University of Toronto, Toronto, Ontario M5S 3E1, Canada; †† Department of Molecular Genetics, University of Toronto, Toronto, Ontario M5G 1A8, Canada; ‡‡ 7268 Institute for Systems Biology, Seattle, Washington 98109, United States; §§ Department of Population Health Sciences, Oakfield House, Oakfield Grove, 1980 University of Bristol, BS8 2BN Bristol, United Kingdom; ∥∥ 8784 University of California San Diego, Skaggs School of Pharmacy and Pharmaceutical Sciences, San Diego, California 92093, United States; ⊥⊥ RECETOX, Faculty of Science, 37748 Masaryk University, Kotlářská 2, 602 00 Brno, Czech Republic; ## mzio GmbH, Altenwall 26, 28195 Bremen, Germany; ∇∇ Program for Bioinformatics, 1846 Boston University, Boston, Massachusetts 02215, United States; ○○ 6426 Agilent Technologies, 5301 Stevens Creek Blvd, Santa Clara, California 95051, United States; ◆◆ San Diego State University, Department of Chemistry and Biochemistry, San Diego, California 92115, United States; ¶¶ Leibniz Institute of Plant Biochemistry, Halle 06120, Germany; ††† German Centre for Integrative Biodiversity Research (iDiv), Halle-Jena-Leipzig 04103, Germany; ‡‡‡ European Molecular Biology Laboratory—European Bioinformatics Institute (EMBL-EBI), Wellcome Genome Campus, Hinxton, Cambridge CB10 1SA, United Kingdom; §§§ Department of Computer Science, University of Toronto, Toronto, Ontario M5G 1A8, Canada; ∥∥∥ 10289 Thermo Fisher Scientific, 355 River Oaks Parkway, San Jose, California 95134, United States; ⊥⊥⊥ European Research Institute for the Biology of Ageing, University Medical Center of Groningen, Groningen 9700 RB, Netherlands; ### 9142 Ruhr University Bochum, Medical Faculty, Medical Bioinformatics, Bochum D-44801, Germany; ∇∇∇ Ruhr University Bochum, Medical Faculty, Core Unit Bioinformatics —CUBiMed.RUB, Bochum D-44801, Germany; ○○○ Department of Computer Science, 8790 University of California Riverside, 900 University Ave. Riverside, California 92521, United States; ◆◆◆ Research and Development, Bruker Belgium nv., Kontich 2550, Belgium; ¶¶¶ Applied Bioinformatics, Dept. of Computer Science, University of Tübingen, Tübingen D-72074, Germany; †††† Institute for Bioinformatics and Medical Informatics, 72076 Tübingen, Germany; ‡‡‡‡ Translational Bioinformatics, University Hospital Tübingen, 72074 Tübingen, Germany; §§§§ OpenMS Inc., Erie, Pennsylvania 16502, United States; 34 BioOrganic Mass Spectrometry Laboratory (LSMBO), IPHC UMR 7178, University of Strasbourg, CNRS, Strasbourg 67000, France; 35 Infrastructure Nationale de Protéomique ProFI-FR2048, Strasbourg 67087, France

**Keywords:** mass spectrometry, standards, data formats, proteomics, metabolomics, lipidomics, proteomics standards initiative

## Abstract

Advances in mass spectrometry (MS) instrumentation, including higher resolution, faster scan speeds, and improved sensitivity, have dramatically increased the data volume and complexity. The adoption of imaging and ion mobility further amplifies these challenges in proteomics, metabolomics, and lipidomics. Current open formats such as mzML and imzML struggle to keep pace due to large file sizes, slow data access, and limited metadata support. Vendor-specific formats offer faster access but lack interoperability and long-term archival guarantees. We here lay the groundwork for mzPeak, a next-generation community data format designed to address these challenges and support high-throughput, multidimensional MS workflows. By adopting a hybrid model that combines efficient binary storage for numerical data and both human- and machine-readable metadata storage, mzPeak will reduce file sizes, accelerate data access, and offer a scalable, adaptable solution for evolving MS technologies. For researchers, mzPeak will support complex workflows and regulatory compliance through faster access, improved metadata, and interoperability. For vendors, it offers a streamlined, open alternative to proprietary formats. mzPeak aims to become a cornerstone of MS data management, enabling sustainable, high-performance solutions for future data types and fostering collaboration across the mass spectrometry community.

## Graphical abstract

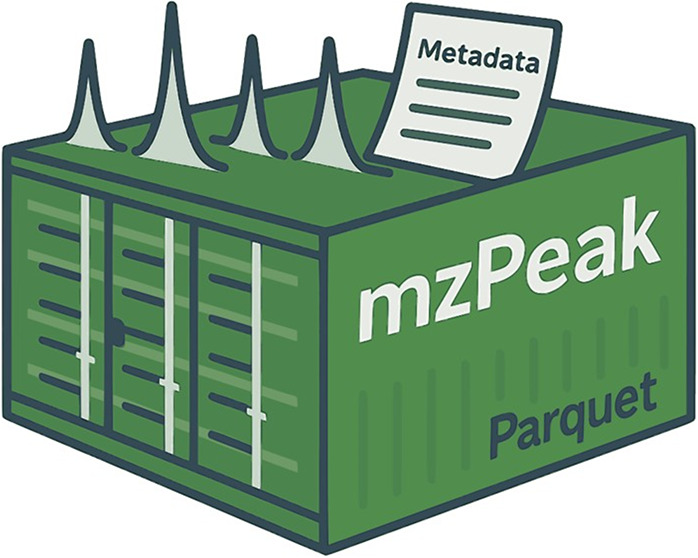

## 1 Introduction

The field of mass spectrometry (MS) has transformed significantly in the past decade, driven by rapid technological advances that have pushed the boundaries of what is possible. Modern mass spectrometers are now capable of generating very large data sets in a fraction of the time required by earlier instruments. These advances create exciting new opportunities in MS-based omics disciplines but also present significant challenges, particularly in the way data is stored and accessed. Currently, there is no universally effective format for efficiently managing and preserving the massive, complex data sets generated by these advanced instruments.

The two main options available today, the open format mzML and vendor-specific formats, both have substantial limitations. mzML,^1,2^ is the widely adopted community-driven standard defined by the Proteomics Standards Initiative of the Human Proteome Organization (HUPO-PSI).^3^ mzML has been a cornerstone of MS data for over a decade, but it was not designed to handle the rapidly increasing scale and complexity of modern data. Its text-based XML structure results in large file sizes and slow data access, making it inefficient for high-throughput workflows. Binary vendor-specific formats, while optimized for their respective instruments, pose challenges of their own. They often require proprietary software for access, lack interoperability, and require the continued availability of legacy vendor software configurations for long-term data preservation, creating barriers for data sharing and reuse. Additionally, evolving regulatory requirements in fields such as precision medicine (e.g., personalized molecular phenotyping) and chemical safety (e.g., environmental and human monitoring programs) are increasingly driving the need for robust solutions for long-term data and metadata storage, capabilities that are inadequately supported by both mzML and vendor-based formats.

This paper introduces mzPeak, a next-generation data format aimed at addressing these challenges. Building on the lessons from mzML and binary formats, mzPeak offers a future-resilient solution tailored for modern MS workflows. It will improve storage efficiency, accelerate data access, and enhance interoperability across platforms and vendors while aligning with evolving regulatory demands. Figure 1 summarizes the key challenges of existing formats and introduces the design principles guiding mzPeak.

**Figure 1:**
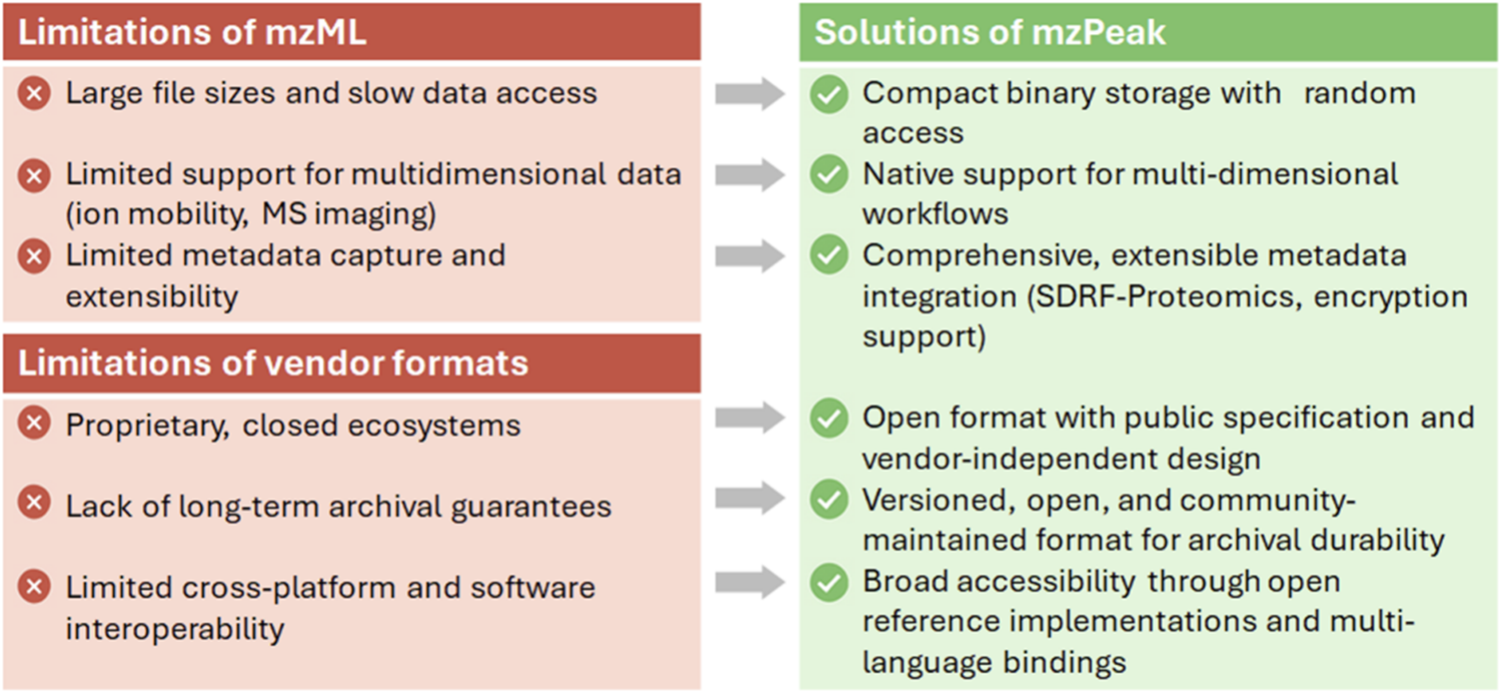
Limitations of existing mass spectrometry data formats and solutions proposed by mzPeak. A comparison of key limitations associated with current open (mzML) and vendor-specific mass spectrometry formats and how mzPeak aims to address these challenges through a compact, interoperable, and extensible design.

## 2 Challenges with Current Formats

### 2.1 Limitations of XML-Based Formats

The development of mzML was a significant milestone in MS data standardization, providing a flexible raw data format that greatly improved data exchange and interoperability at the time of its introduction. Designed as an XML-based format to be both human- and machine-readable, mzML marked a departure from vendor-specific, proprietary formats that required specialized, often limited-access software to read. It addressed many challenges faced by the MS community over a decade ago and has since served as a cornerstone for MS data handling. However, the landscape has evolved dramatically, and mzML’s text-based XML structure now introduces inefficiencies that are increasingly problematic in today’s high-throughput, multidimensional workflows.

One of mzML’s design bottlenecks is that it is a text-based XML format, which makes file sizes much larger than necessary (see the Supporting Information for further technical discussion). During the development of mzPeak, the coordinating committee conducted a survey of users of mzML. The results indicated that the file size expansion is a significant burden, particularly in laboratories generating data in the terabyte range per instrument each month, where researchers are often expected to store this data for over 10 years or even indefinitely. Additionally, users rely increasingly on various cloud storage solutions, highlighting the need for open standards that are compatible with different storage options and support affordable, efficient, and long-term data storage.

Moreover, mzML’s reliance on XML makes it suboptimal for high-throughput environments where rapid data access is critical. Parsing large XML files is computationally expensive, and as data sets grow in size and complexity, this becomes a limiting factor in processing speed. Responding to these limitations, some computational groups have developed nonstandard binary formats as intermediate data structures (Supporting Table 1), but these formats lack interoperability, further complicating data management. The growth of mass spectrometry imaging (MSI, also known as imaging mass spectrometry) has underscored the need for a unified format that can meet its unique requirements, including large file sizes, spatial coordinate tracking, efficient access to both spectra and ion images, support for ion mobility, and the potential inclusion of MS/MS or triple-quadrupole data.

Researchers working with high-resolution instruments, multidimensional ion mobility workflows, or MSI data are often hindered due to software supporting only part of the capabilities of their mass spectrometers. This leads to some software not being able to load the files due to the memory requirements and other software having inefficient data access due to the legacy data structures used in the XML-based formats. Ultimately, these barriers limit the potential of MS applications. This is particularly important for users without in-depth knowledge and experience in programming who rely on existing software implementations. Furthermore, a survey of mass spectrometrists reported that difficulty finding a well-advertised, complete, and/or documented implementation of mzML and/or imzML,^4^ a similar format for MSI data, had affected their decision to use mzML and/or imzML and created problems both for the users as well as for software developers. This emphasizes the need for robust support and documentation in future formats.

### 2.2 Limitations of Vendor-Specific Formats

In contrast to mzML, vendor-specific formats are optimized for the performance of the instruments they support, tailoring data access and storage to specific instrumentation, acquisition parameters, and diagnostic metadata. However, these proprietary formats often lack long-term archival considerations and limit interoperability. Access to vendor formats typically requires software libraries with restrictive licenses. Even when vendor software development kits (SDKs) are freely available, they often depend on specific platforms, operating systems, or programming languages, making long-term data access uncertain. While vendor SDKs have improved access to proprietary data, they do not fully address the needs for open, long-term archival, regulatory compliance, and reproducible science across diverse software ecosystems. This restriction on accessibility poses significant risks to data preservation over time. In addition, for an academic developer of software, it creates an additional burden to support software formats from multiple vendors, and software distribution is hindered by vendors’ license restrictions (e.g., redistribution restrictions).

By comparison, a unified community-supported format helps to abstract from this complexity, and open formats facilitate reproducible science by ensuring long-term, license-free, single-entry-point, and system-agnostic data access, all of which are indispensable for scientific reproducibility. A universal open format such as mzPeak would therefore be essential to support seamless data exchange and reproducibility across diverse mass spectrometers, addressing these limitations effectively.

### 2.3 Lessons Learned from mzMLb and Other Binary Formats

The development of mzMLb^5^ represented an attempt to address some of the limitations of mzML, particularly around data compression and access speed (Supporting Table 1). By using HDF5, a binary storage format, mzMLb reduced file sizes and improved data retrieval times, making it a more efficient option for handling large data sets. However, despite these technical improvements, mzMLb did not gain widespread adoption. One critical issue arising from the mzML specification, left unresolved by the conversion process, was the limited availability of metadata. This hindered its usability in workflows that depend on detailed experimental information, such as multidimensional and regulatory-driven studies. The lack of metadata integration reduced its usability, as researchers found it challenging to store and manage essential contextual information within mzMLb just as it was within mzML. Because the conversion relied entirely on the limited metadata exposed by vendor libraries and the implementation(s) were complex to modify, resolving this limitation within the MS data ecosystem was not practical. Furthermore, the community perceived mzMLb as offering limited additional value over mzML, as it did not address broader needs such as vendor interoperability or regulatory compliance, while greatly increasing the technical burden to support it. These lessons emphasize the need for mzPeak to offer both improved data handling efficiency and robust metadata support to ensure broad utility and adoption in the MS community.

## 3 Our Vision for mzPeak

### 3.1 Scalable, Open Solution

mzPeak is designed to overcome the limitations of the aforementioned formats by adopting a hybrid model that combines efficient binary storage for numerical data and human-readable metadata storage. This hybrid approach ensures efficient storage and faster read/write times, making it well-suited for complex data sets generated by high-resolution, multidimensional workflows such as ion mobility spectrometry and MSI. For initial implementation studies, we will use Parquet (https://parquet.apache.org/), an open source, data file format designed for efficient storage and retrieval, including compression and encoding schemes to ensure scalability without sacrificing flexibility. Parquet access is available for many programming languages.

The format will use the HUPO-PSI Mass Spectrometry controlled vocabulary (PSI-MS),^6^ which ensures that mzPeak will align with widely accepted terms and definitions. Designed with a native binary format, mzPeak will enable random access to spectra, chromatograms, ion images, and mobilograms, ensuring fast and efficient data retrieval. Additionally, it will allow for lossless interconversion with vendor-based formats, preserving data integrity while ensuring compatibility across platforms. Released under an open license and free of patent restrictions, mzPeak will be designed to support both the immediate and future needs of the MS community, offering a robust foundation for managing the growing complexity of MS data.

### 3.2 Comprehensive Metadata

One of the critical shortcomings of older formats is their limited ability to store and annotate comprehensive metadata, particularly at sample and MS run levels. mzPeak will address this limitation by enabling detailed annotation of both sample characteristics and mass spectrometer configurations, while integrating the community-supported metadata standard SDRF-Proteomics,^7^ enabling detailed annotation of both sample characteristics and mass spectrometer configurations. This includes crucial information, such as experimental conditions, run-specific parameters, and sample descriptions, ensuring the data can be fully utilized and interpreted across different MS platforms from various vendors.

By combining sample-level metadata with operational details, such as pump pressures across an LC gradient (even when not directly retrievable from vendor MS files), mzPeak will support seamless metadata annotation and export. These capabilities are essential for ensuring data comparability in public repositories and for meeting regulatory requirements, where complete and accurate metadata are critical for long-term usability and integrity.

Vendor raw files often contain embedded instrument-specific metadata, which may have privacy and/or IP implications. In clinical settings, there is a potential need for storage of Personally Identifiable Information on clinical samples. To address these needs, mzPeak will support the storage of binary objects and encrypted metadata fields, building on mechanisms such as column-level encryption and page-level checksumming available in Parquet.

### 3.3 Flexible and Future-Resilient Design

mzPeak will not just be a solution for today’s challenges but will be designed to accommodate the future evolution of MS approaches. It should have a flexible yet machine-readable structure that allows for the incorporation of new data types and workflows, ensuring that the format remains relevant, even with ongoing technological advances. It should be able to support recently emerged and still-evolving modes of data acquisition such as MSI and single-cell mass spectrometry analyses where the experimental setup and data structures can substantially differ from more traditional chromatography-based mass spectrometry used in bulk proteomics, metabolomics, or lipidomics. Therefore, this format can evolve alongside the MS field, supporting new analytical techniques, instrumentation, and data analysis workflows. Extensive discussions within the mzPeak committee established the need to recognize that this new format needs to balance between minimizing the size of the files and minimizing access times. In recognition of the differing needs of different members of the community, technical development will explore these trade-offs and present possible compromises back to the committee.

To further ensure long-term sustainability, mzPeak builds on technologies and frameworks with demonstrated community support and institutional stability. The controlled vocabularies maintained by the Proteomics Standards Initiative (HUPO-PSI),^3^ established in 2002,^8^ and the PSI-MS ontology, maintained since 2013,^6^ provide a mature, community-governed foundation for metadata standardization. For serialization, mzPeak will be prototyped in Apache Parquet (https://parquet.apache.org/), a top-level project of the Apache Software Foundation launched in 2013, which is widely adopted across diverse industries and offers strong guarantees of continued maintenance and interoperability. Together, these choices support mzPeak’s goal of delivering a format that remains durable, extensible, and accessible as mass spectrometry technologies continue to evolve.

## 4 Benefits of mzPeak

### 4.1 For Researchers

mzPeak is being developed to address the key limitations of both existing community standards and proprietary vendor formats. Compared with mzML, mzPeak aims to offer faster data access and smaller file sizes by using efficient binary storage for numerical data, enabling quicker and more scalable analysis workflows. Compared to vendor-specific formats, mzPeak seeks to improve interoperability across platforms and software ecosystems by adopting an open, standardized structure, facilitating seamless data sharing, and reducing dependency on proprietary tools.

Interoperability is another major advantage. mzPeak will facilitate seamless data sharing between software platforms, breaking down barriers to compatibility and enabling more flexible data analysis. At the data level, proteomics, metabolomics, and lipidomics share substantial similarities apart from aspects such as polarity, with differentiation largely driven by analytical and sample preparation techniques. This commonality ensures that mzPeak can serve these fields effectively while remaining adaptable to emerging -omics fields. Moreover, its interoperability is particularly beneficial for multiomics studies, where the integration of data from proteomics, metabolomics, and lipidomics is critical for providing a comprehensive view of biological systems.

Long-term data preservation is crucial in fields such as precision medicine, regulatory monitoring programs, and MS library building. Preserving original raw data enables future researchers to reprocess and validate spectra as instrumentation and algorithms evolve. mzPeak was designed with archival durability in mind, providing a stable and secure format that aligns with regulatory requirements for data retention and accessibility. By supporting robust metadata and standardized data structures, mzPeak will ensure that critical information remains intact and accessible, even as technologies and analytical platforms evolve. This guarantees that data generated today will remain interpretable well into the future, enabling longitudinal studies and regulatory reviews without the risk of data degradation or incompatibility.

Additionally, mzPeak’s ability to present all available vendor raw data, unlike current standards that often omit or fail to store certain instrument-specific details, makes it a more complete and efficient solution. This, combined with its open design, reduces the burden on institutional or public data repositories such as EMBL-EBI’s PRIDE,^9^ MassIVE, Metabolomics Workbench, and MetaboLights,^10^ contributing to more sustainable long-term storage and archiving practices. A side-effect of data submission in mzPeak would be easier access for web services to the majority of MS data in an archive, instead of only those projects that deposit open formats alongside vendor raw files.

### 4.2 For Vendors

For MS vendors, mzPeak offers strategic advantages beyond just technical performance. The increasing focus on regulatory compliance, particularly in fields such as precision medicine (e.g., personalized molecular phenotyping), requires auditable data formats that can be archived for long periods while remaining accessible and usable and with an assurance of integrity. mzPeak addresses this need by providing a format that ensures both long-term data preservation and rapid access when needed.

By adopting an open standard, vendors can also reduce the costs associated with maintaining proprietary data formats. The transition to mzPeak allows vendors to focus on their core innovations while leveraging a community-driven standard for routine data management tasks.

Software vendors have substantial incentives to adopt mzPeak. Using an open format saves time by only requiring a single implementation in order to be able to read data from any instrument that can output to mzPeak. In comparison to mzML, mzPeak allows efficient storage and access patterns to data generated in a wider variety of experiment types, including imaging MS, and complex ion mobility experiments. Additionally, using Parquet, mzPeak enables seamless storage of MS data in cloud environments.

### 4.3 For Regulatory Agencies

mzPeak will be designed to meet the needs of regulatory agencies responsible for data integrity, auditability, and long-term accessibility. By developing a versioned, openly documented format with structured metadata and encryption support for sensitive information, mzPeak has potential with regulatory frameworks such as those used in clinical trials. The use of an open, community-driven data format will facilitate reproducible analyses and reliable data interpretation without a dependence on proprietary software. This would, therefore, help regulatory bodies to confidently evaluate data submissions.

## 5 Roadmap for mzPeak Development

### 5.1 Community-Driven Collaboration

The development and adoption of mzPeak will rely on the active collaboration between the MS community and key stakeholders. Formed to address the need for a new open file format, the mzPeak Committee has focused on archival and reanalysis needs for large-scale omics studies. The Committee has held roundtable discussions at key conferences and conducted a community survey to gather insights. Moving forward, the Committee will continue to collaborate with researchers, instrument vendors, and software developers to ensure that mzPeak aligns with the diverse needs of the MS ecosystem. HUPO-PSI, with its history of fostering community standards, will play a critical role as well in guiding this development, with inclusion of other communities representing metabolomics, lipidomics, MSI, and single-cell MS. The current mzPeak proposal has received early interest and constructive feedback from researchers, leadership, and developers from key software packages, and several instrument vendors, reflecting a shared recognition of the need for an improved open format and a willingness to help shape its development. To further broaden this effort, the mzPeak Committee is open to new contributors, researchers, developers, or stakeholders interested in helping to develop or support the format, and they are encouraged to reach out and join the initiative.

### 5.2 Technical Implementation

mzPeak will be released with a reference implementation in a low level programming language (e.g., C++) with bindings for multiple programming languages (e.g., Python), to ensure broad accessibility for both researchers and vendors. To prevent issues like those seen with earlier formats, the development process will explicitly include a roadmap for a well-advertised, fully functional reference implementation from the outset. This implementation will include a validator to ensure compatibility with the mzPeak standard and facilitate seamless integration across different operating systems through cross-platform interoperability.

The data storage model will be natively binary, allowing for random access to spectra, chromatograms, ion images, and mobilograms, ensuring efficiency in handling large and complex data sets. Vendors will also be able to store essential technical metadata, including but not limited to MS and LC settings and run-specific parameters. Each run will be stored as a single, self-contained file, providing a comprehensive archive of all of the data and metadata.

Additionally, the reference implementation will be designed to integrate with existing tools, minimizing disruptions to established workflows by providing converters, backends, or wrappers. This approach addresses one of the shortcomings of previous standards by prioritizing compatibility and encouraging adoption through developer-friendly tools and clear documentation. Selection of serialization technologies explicitly considers long-term stability, including the availability of multiple independent implementations and the sustainability of library maintenance over time.

To ensure long-term viability, mzPeak will adopt clear versioning practices and maintain openly accessible documentation for each format release. Particular attention will be given to preserving the stability of core data structures, such as indexing schemes and metadata organization, across future versions. As technology continues to evolve, we aim to mitigate risks by maintaining open documentation practices, archiving format specifications publicly, and fostering ongoing community engagement. These efforts aim to help ensure that data generated today remain accessible and usable for future research, clinical, and regulatory needs.

All technical comments collected before the submission of this paper are summarized in the Supporting Notes.

### 5.3 Adoption Strategy

The adoption of mzPeak will be driven by both its technical advantages and its strategic collaborations with key stakeholders. To ensure broad acceptance, strategic partnerships will be established not only with major MS vendors but also with developers of existing “keystone” software, e.g., OpenMS,^11^ ProteoWizard,^12^ METASPACE,^13^ public data repositories, prominent academic institutions, and influential users who can advocate for the format’s adoption. Regulatory agencies will also play a critical role by encouraging or requiring data deposits in standardized formats, such as mzPeak for compliance purposes.

An additional enabler of adoption will be the availability of intuitive GUI-based tools for data inspection and exploration. While generic Parquet viewers exist, they are not tailored to mass spectrometry and cannot support spectral, chromatographic, or imaging-based visualization. To ensure accessibility for nonprogrammers and wet-lab scientists, mzPeak adoption will benefit from user-friendly software similar to mzmine^14^ or vendor-specific viewers. These tools will allow researchers to perform quality control, inspect metadata, and explore the file content.

Informed by lessons learned from earlier formats, such as mzML, we also recognize that successful uptake of mzPeak will depend on a well-coordinated launch. To support this, mzPeak will be released with clear documentation, example files, and language bindings. A strong emphasis will be placed on messaging and visibility, including tutorials, community outreach, and close coordination with developers of widely used tools. These efforts are intended to lower the barrier to adoption and support a smooth transition for both new and experienced users.

This multifaceted approach positions mzPeak as the default option for data storage and analysis. The goal is to create a format that is technically superior, aligned with regulatory and community needs, and widely supported across the MS community.

## 6 Conclusions

As mass spectrometry continues to evolve, so too do the tools we use to manage the data it generates. mzPeak is designed to address the challenges of today’s high-throughput data environments while preparing the field for future advancements. By building on the lessons of mzML, mzMLb, imzML, and others, mzPeak strives to offer a scalable, future-resilient solution that benefits researchers, vendors, and regulators. We invite researchers, developers, instrument vendors, and other members of the mass spectrometry community to contribute their expertise and perspectives as we finalize the initial specifications and begin prototyping reference implementations with the goal of aligning mzPeak with the practical needs of current and future MS workflows.

## Supplementary Material



## Notes

Limitations of XML-based formats such as mzML, and design considerations for mzPeak, including the use of Parquet as an initial serialization format; comparison of existing interchange formats (Table S1) (PDF)
