# Supplementary material for: mzPeak: Designing a Scalable, Interoperable, and Future-Ready Mass Spectrometry Data Format
Source: J Proteome Res. 2025 Oct 2;24(11):5329–35. doi: 10.1021/acs.jproteome.5c00435 (PMC12604042; doi:10.1021/acs.jproteome.5c00435)
Supplement: Supplementary file 1 [file pr5c00435_si_001.pdf]

mzPeak: designing a scalable, interoperable, and future-ready mass spectrometry data format

Tim Van Den Bossche[1,2], Theodore Alexandrov[3,4], Aivett Bilbao[5,6], Wout Bittremieux[7], Federico Ivan Brigante[8], Matthew Chase Chambers[9], Joshua Charkow[10,11], Eric Deutsch[12], Andrew W. Dowsey[13], Yasin El Abiead[14], Ralf Gabriels[1,2], Helge Hecht[15], Steffen Heuckeroth[16], Joshua A. Klein[17], Michael Knierman[18], Lennart Martens[1,2], Robert L. Moritz[12], Laura-Isobel McCall[19], Steffen Neumann[20,21], Yasset Perez-Riverol[22], Hannes L. Röst[10,11,23], Elliott J. Price[15], Jim Shofstahl[24], David L. Tabb[25], Julian Uszkoreit[26,27], Juan Antonio Vizcaíno[22], Mingxun Wang[28], Sander Willems[29], Dirk Winkelhardt[26,27], Oliver Kohlbacher[30,31,32,], and Samuel P. Wein[30,33]*

** Corresponding author: Oliver Kohlbacher (oliver.kohlbacher@uni-tuebingen.de)*

(1) Department of Biomolecular Medicine, Faculty of Medicine and Health Sciences, Ghent University, 9052 Ghent, Belgium
(2) CompOmics, VIB - UGent Center for Medical Biotechnology, VIB, 9052 Ghent, Belgium
(3) Department of Pharmacology, University of California San Diego, La Jolla, CA 92093, USA
(4) Department of Bioengineering, University of California San Diego, La Jolla, CA 92093, USA
(5) Environmental Molecular Sciences Laboratory, Pacific Northwest National Laboratory, Richland, WA, 99352, USA
(6) US Department of Energy Agile BioFoundry, Emeryville, CA, 94608, USA
(7) University of Antwerp, 2020 Antwerpen, Belgium
(8) Institute of Organic Chemistry and Biochemistry of the Czech Academy of Sciences, 160 00 Prague, Czech Republic
(9) University of Washington, 98195 Seattle, Washington
(10) Donnelly Centre for Cellular and Biomolecular Research, University of Toronto, Toronto, Ontario M5S 3E1, Canada
(11) Department of Molecular Genetics, University of Toronto, Toronto, Ontario M5G 1A8, Canada
(12) Institute for Systems Biology, Seattle, Washington, 98109, United States
(13) Department of Population Health Sciences, Oakfield House, Oakfield Grove, University of Bristol, BS8 2BN
(14) University of California San Diego, Skaggs School of Pharmacy and Pharmaceutical Sciences, San Diego, CA, USA
(15) RECETOX, Faculty of Science, Masaryk University, Kotlářská 2, 602 00 Brno, Czech Republic
(16) mzio GmbH, Altenwall 26, 28195 Bremen, Germany
(17) Program for Bioinformatics, Boston University, Boston, Massachusetts 02215, United States
(18) Agilent Technologies, 5301 Stevens Creek Blvd, Santa Clara, CA 95051, United States
(19) San Diego State University, Department of Chemistry and Biochemistry, San Diego, CA, USA
(20) Leibniz Institute of Plant Biochemistry, Halle, Germany
(21) German Centre for Integrative Biodiversity Research (iDiv) Halle-Jena-Leipzig, Germany
(22) European Molecular Biology Laboratory - European Bioinformatics Institute (EMBL-EBI), Wellcome Genome Campus, Hinxton, Cambridge, United Kingdom
(23) Department of Computer Science, University of Toronto, Toronto, Ontario M5G 1A8, Canada
(24) Thermo Fisher Scientific, 355 River Oaks Parkway, San Jose, CA 95134, United States
(25) European Research Institute for the Biology of Ageing, University Medical Center of Groningen, Groningen, Netherlands
(26) Ruhr University Bochum, Medical Faculty, Medical Bioinformatics, Bochum, D-44801, Germany
(27) Ruhr University Bochum, Medical Faculty, Core Unit Bioinformatics - CUBiMed.RUB, Bochum, D-44801, Germany
(28) Department of Computer Science, University of California Riverside, 900 University Ave. Riverside CA 92521
(29) Research and Development, Bruker Belgium nv., Kontich, Belgium
(30) Applied Bioinformatics, Dept. of Computer Science, University of Tübingen, Tübingen, Germany
(31) Institute for Bioinformatics and Medical Informatics, 72076 Tübingen, Germany
(32) Translational Bioinformatics, University Hospital Tübingen, 72074 Tübingen, Germany
(33) OpenMS inc., Erie, PA 16502, USA

Table of contents

Supplementary Notes...2
Supplementary Table S1...5

Supplementary notes

Limitations of XML-based formats

XML repeats structural information explicitly while offloading semantics to a shared schema, instead of both structure and meaning, to provide a degree of human readability. XML is also inefficient for storing large numerical datasets, either using two to ten times more space to encode 32-bit float imprecisely but human readable, or using 33% more space to base64 encode the numerical bytes exactly. Even with optional compression, mzML files[1] still end up being four to eighteen times larger than the original proprietary formats[2] but also prevents random access to the data using commonly available tools.

A scalable, open solution

One of the core developments for mass spectrometry (MS) in recent years has been the addition of further dimensions of separation or analysis. The former is seen in the explosive growth of ion-mobility data and the latter is seen in the rise of MS imaging (MSI). Looking to the future, mzPeak needs to be designed in such a way that future developments that would further increase the dimensionality of our data do not break or indeed require additions to the format itself.

There is still active discussion as to what format mzPeak will use for serialization. Parquet is mentioned by name in this document as it is the serialization format that the technical committee has decided to use for our initial proof of concept code. In choosing what serialization format we are going to use for mzPeak, we need to keep in balance the specificity of the library to handle the data types that we are working with, versus the level of general support that the format has in the wild. Here we discuss the pros and cons of Parquet, and alternative options that we have considered so far.

Parquet, for example, is a good choice, as there are lots of use cases for it across a wide variety of different areas of data science, and as a result, there are lots of supporting implementations out there. There are at least three different independent compatible reference implementations in C++, Java, Rust, and it's good at interoperability with other languages.

The predicted longevity of the container format is also important. We need to make sure that whatever decision we make today, there is going to be continued maintenance on that library through the predicted lifespan of mzPeak. Again, using Parquet as an example, Parquet has good support through the Apache Foundation, which is indicative of good long-term survivability.

Another consideration is accessibility; because Parquet is implemented across multiple languages, it's easier for users to read the raw container file, and therefore easier for us to implement a reader on top of it. Compared to a case like HDF5 where if you didn't have access to the C library because A) no C ABI or B) can't use the must-have plugins because of issues with linking stage of compilation, an implementer may have to try to use a partial reimplementation of the format with 8 different kinds of strings.

On the other hand Parquet is not obviously the best solution from a strictly technical perspective. Parquet is a column-based data format, so what are the columns here? Instead, some multi-dimensional tensor format seems appropriate, particularly for the raw data where we have at least two arrays or dimensions per spectrum and often more with ion mobility.

A tensor is just a strided/nested array with more than two dimensions. Nothing stops us from representing one as either an in-row list or an unzipped long table with all the metadata with run-length encoding (RLE). There are costs associated with either decision, there's no free lunch after all, but this price is paid either once per dimension per row (wide), or once per run group per page (long). These choices each introduce trade offs that impact how the file would be read, and how different tools might interact with them. The same constraints apply to the tensor format when it comes time for compression and storage, but it may be able to make certain assumptions during the layout of data in the byte stream to reduce the costs in space or access time. These are examples of more general array storage formats which, due to the recentness of their emergence in the field, have only a single implementation, often governed by a single institution or corporation, and may not even have any formal guarantee of stability of the container file format. For example, DuckDB's file format would satisfy most of the same needs that Parquet would while adding many other desirable capabilities like multiple logical tables per file and search indices, only recently announced their serialization format would be stable going forwards, but there remains only the one canonical implementation of the reader written by DuckDB themselves which they provide bindings to for most major languages. We greatly hope that DuckDB will be with us to welcome the year 2050 along with SQLite, but there are no guarantees.

Parquet's main weaknesses are granular random access and storage heterogeneity. Storage heterogeneity is usually addressed with multiple files or by schema partitioning. Schema partitioning here means each row in the table is a structure containing *n* distinct types of nested nullable structures, one for each type of entity we wish to store, e.g. spectrum, chromatogram, custom index block, et. cetera, and then organize the file so that most rows containing the same type of substructure are grouped together for RLE to compress away the other branches. While our hope is that we can achieve our goals while storing all data in a single file, it may be necessary to split a Parquet file across ordering dimensions to achieve the desired performance as Parquet does not itself encode out-of-stream sort indices. Random access is the bit that can't be fixed without ahead-of-time planning on the schema, the sorting order, and expected anticipation of access patterns. Parquet uses blocked and compressed storage, which means that in order to read a single value, all the values in the block must be decompressed together. The smaller the block, the less efficient the compression but the faster that block can be decompressed on average. While block sizes can be tuned, there are limits and they cannot be configured granularly per column. The canonical way to address this problem with Parquet is to split (and sort) files so that you are more likely to want to read whole blocks at a time, and so that block-level statistics make it easier to filter out blocks entirely. This is undesirable for a format intended to be uploaded/downloaded with conventional web browsers that cannot operate on directories as a unit.

Exactly what's in the schema is undecided yet. We could end up mirroring the mzML schema very easily with a few recurring CVparams burned in as columns, or something resembling mzTab, depending upon what works best. Many facets of the mzML schema were designed for use cases that never emerged, or required a degree of nesting that was not useful in

practice. Any new schema is going to need to be suitable to access along different dimensions, in traditional mzML data could be stored in spectra and chromatograms, since we want to allow for storage of imaging data, and ion-mobility data, the schema also will need to allow for access across spatial dimensions and ion-mobiliograms. There has been some discussion of using peaks as the base unit of storage, and treating each peak as a row in the Parquet table, with columns corresponding to M/Z, intensity, and whatever separate dimensions of separation along which the data was acquired. The ordering of these rows would be formally arbitrary, with indexing in separate tables to reconstruct the underlying scans, chromatograms, mobilograms, or pixels. We are currently doing technical implementation studies to determine how feasible this approach is, and what the best way to store co-occurring metadata is.

Parquet as a container format checksums each data page for integrity checking and granular file encryption to protect sensitive information and prevent tampering.
https://parquet.apache.org/docs/file-format/data-pages/checksumming/

https://parquet.apache.org/docs/file-format/data-pages/encryption/

Supplementary Table 1. Comparison of existing interchange formats

Format Name	Container Format	Implementations	Data-Metadata disposition	Metadata Format	Ion Mobility Compatibility	Uses mzML Data Model	Reference
mzML	XML	Multiple (https://www.psidev.info/mzml)	In-band	XML Text	Compatible	Yes	[1]
mzMLb	HDF5	C++, Python, Rust, R (via C++)	Out-of-band	XML Text	Compatible	Yes	[2]
mz5	HDF5	C++, R (via C++)	In-band	Binary Structures	Not compatible	Yes	[3]
imzML	XML + Custom binary (two different files)	C++, R, Python, Java	Out-of-band	XML Text	Not compatible	No	[4]
mzDB	SQLite3	C++, Java	In-band, Replicated out-of-band Index	XML Text	Not compatible	Yes	[5]
Toffee	HDF5	C++, Python	Out-of-band	XML Text	Required	No	[6]
mzA	HDF5	Python	Out-of-band	Binary Structures	Compatible	No	[7]

StackZDPD	Custom binary + JSON	C#, Java	Out-of-Band	JSON	Compatible	No	[8]
mzTree	Custom binary (R-Tree based)	Java	In-Band	SQLite	Not compatible	No	[9]
mzXML	XML	Python, C++	In-Band	XML Text	Not compatible	No	[10]

Supplementary References

1. Lennart Martens, Matthew Chambers, Marc Sturm, Darren Kessner, Fredrik Levander, Jim Shofstahl, Wilfred H. Tang, et al. “mzML–a Community Standard for Mass Spectrometry Data.” *Molecular & Cellular Proteomics: MCP* 10, no. 1 (January 2011): R110.000133. https://doi.org/10.1074/mcp.R110.000133.
2. Ranjeet S. Bhamber, Andris Jankevics, Eric W. Deutsch, Andrew R. Jones, and Andrew W. Dowsey. “mzMLb: A Future-Proof Raw Mass Spectrometry Data Format Based on Standards-Compliant mzML and Optimized for Speed and Storage Requirements.” *Journal of Proteome Research* 20, no. 1 (January 1, 2021): 172–83. https://doi.org/10.1021/acs.jproteome.0c00192.
3. Mathias Wilhelm, Marc Kirchner, Judith A. J. Steen, and Hanno Steen. “Mz5: Space- and Time-Efficient Storage of Mass Spectrometry Data Sets *.” *Molecular & Cellular Proteomics* 11, no. 1 (January 1, 2012). https://doi.org/10.1074/mcp.O111.011379.
4. Thorsten Schramm, Zoë Hester, Ivo Klinkert, Jean-Pierre Both, Ron M. A. Heeren, Alain Brunelle, Olivier Laprévote, et al. “imzML–a Common Data Format for the Flexible Exchange and Processing of Mass Spectrometry Imaging Data.” *Journal of Proteomics* 75, no. 16 (August 30, 2012): 5106–10. https://doi.org/10.1016/j.jprot.2012.07.026.
5. David Bouyssié, Marc Dubois, Sara Nasso, Anne Gonzalez de Peredo, Odile Burlet-Schiltz, Ruedi Aebersold, and Bernard Monsarrat. “mzDB: A File Format Using Multiple Indexing Strategies for the Efficient Analysis of Large LC-MS/MS and SWATH-MS Data Sets.” *Molecular & Cellular Proteomics: MCP* 14, no. 3 (March 2015): 771–81. https://doi.org/10.1074/mcp.O114.039115.
6. Brett Tully. “Toffee - a Highly Efficient, Lossless File Format for DIA-MS.” *Scientific Reports* 10, no. 1 (June 2, 2020): 8939. https://doi.org/10.1038/s41598-020-65015-y.
7. Aivett Bilbao, Dylan H. Ross, Joon-Yong Lee, Micah T. Donor, Sarah M. Williams, Ying Zhu, Yehia M. Ibrahim, Richard D. Smith, and Xueyun Zheng. “MZA: A Data Conversion Tool to Facilitate Software Development and Artificial Intelligence Research in Multidimensional Mass Spectrometry.” *Journal of Proteome Research* 22, no. 2 (February 3, 2023): 508–13. https://doi.org/10.1021/acs.jproteome.2c00313.
8. Jinyin Wang, Miaoshan Lu, Ruimin Wang, Shaowei An, Cong Xie, and Changbin Yu. “StackZDPD: A Novel Encoding Scheme for Mass Spectrometry Data Optimized for Speed and Compression Ratio.” *Scientific Reports* 12, no. 1 (March 30, 2022): 5384. https://doi.org/10.1038/s41598-022-09432-1.
9. Kyle Handy, Jebediah Rosen, André Gillan, and Rob Smith. “Fast, Axis-Agnostic, Dynamically Summarized Storage and Retrieval for Mass Spectrometry Data.” *PloS One* 12, no. 11 (2017): e0188059. https://doi.org/10.1371/journal.pone.0188059.
10. Patrick Pedrioli, Jimmy Eng, Robert Hubley *et al.* A common open representation of mass spectrometry data and its application to proteomics research. *Nat Biotechnol* **22**, 1459–1466 (2004). https://doi.org/10.1038/nbt1031
